# Giant Cell Tumor of Bone: Biology, Pathophysiology, and Histopathology in the Era of H3F3A

**DOI:** 10.3390/biomedicines14020449

**Published:** 2026-02-17

**Authors:** Bruno Daniel Carneiro, Susana Brilhante, Carlos Silva Faria, Sara Fonseca, Daniel Humberto Pozza

**Affiliations:** 1 Unit of Experimental Biology, Department of Biomedicine, Faculty of Medicine, University of Porto, 4200-319 Porto, Portugal; bcarneiro@med.up.pt (B.D.C.); susanabrilhantx@gmail.com (S.B.); 2 Rheumatology Service, Unidade Local de Saúde do Alto Minho, Hospital Conde de Bertiandos, 4990-078 Ponte de Lima, Portugal; 3 Department of Surgery and Physiology, Faculty of Medicine, University of Porto, 4200-319 Porto, Portugal; carlosafaria@gmail.com (C.S.F.); saracunhafonseca@gmail.com (S.F.); 4 Anaesthesiology Department, São João University Hospital Centre, 4200-135 Porto, Portugal; 5 Institute for Research and Innovation in Health and IBMC, University of Porto, 4200-135 Porto, Portugal

**Keywords:** giant cell tumor of bone, bone neoplasms, H3F3A protein, osteoclasts, tumor microenvironment, osteolysis, denosumab, rankl, osteoclastogenesis, histopathology

## Abstract

Giant cell tumor of bone (GCTB) is a distinctive, intermediate-grade primary bone neoplasm defined by a neoplastic mesenchymal stromal compartment and a prominent osteoclast-rich microenvironment. Although histologically benign, GCTB is clinically consequential due to its locally destructive behavior, propensity for recurrence, and rare capacity for metastasis and malignant transformation. Over the past decade, the identification of recurrent H3F3A p.G34 mutations has fundamentally reshaped the understanding of GCTB pathogenesis, establishing the stromal cell as the true neoplastic driver and positioning the tumor as a paradigmatic epigenetically driven osteolytic disease. This narrative review focus on biology, pathophysiology, and histopathology in the era of H3F3A. H3F3A-mutant stromal cells orchestrate pathological osteoclastogenesis through dysregulated RANKL signaling and chromatin reprogramming, giving rise to the characteristic cellular admixture of osteoclast-type giant cells, mononuclear histiocytic cells, and neoplastic stromal elements. The targeted inhibition of osteoclast activity with denosumab has transformed clinical management, inducing profound morphological changes and bone formation. However, its long-term impact on local control, recurrence patterns, and malignant progression remains unclear. Collectively, GCTB exemplifies a molecularly defined bone tumor in which advances in epigenetic biology and tumor–microenvironment interactions have directly influenced diagnostic practice and therapeutic strategy. Ongoing challenges include refining risk stratification, optimizing treatment sequencing, and clarifying the biological consequences of sustained osteoclast suppression.

## 1. Introduction

Giant cell tumor of bone (GCTB) is a primary bone neoplasm defined by the presence of mononuclear neoplastic stromal cells and numerous reactive osteoclast-type giant cells. Its incidence is approximately 1.8 cases per million per year in Western populations, being higher in Asia, possibly reflecting genetic or environmental factors or differences in diagnostic classification [1,2,3,4]. In fact, while demographic factors such as population age structure and access to specialized diagnostics partially explain these differences, emerging evidence suggests that integrating molecular pathology with epidemiological data may uncover population-specific biological or genetic susceptibilities that influence tumor development and clinical behavior [5,6]. GCTB accounts for approximately 4 to 10% of all primary bone tumors [7,8,9] and affects predominantly adults aged between 20 and 45 years [1,10]. The tumor is slightly predominant in females [1,10,11,12]. Typical symptoms can include the insidious onset of localized pain, swelling and tenderness, decreased range of motion, mechanical symptoms when the tumor is near joints, and pathological fractures [10,13].

The biological nature of GCTB remained debated for decades due to the mixture of reactive multinucleated giant cells and mononuclear cells [14,15,16]. Although histologically benign, the tumor is locally aggressive and capable of pulmonary metastasis [17,18,19,20], placing it in the category of “intermediate locally aggressive” according to the World Health Organization (WHO) [21,22]. The tumor’s clinical course is unpredictable, and despite lacking intrinsic metastatic biology, a small percentage of cases disseminate to the lung. Local aggressiveness and recurrence rates remain significant, particularly after intralesional procedures [7,18,20]. In fact, recurrence rates after surgical curettage may reach 30 to 50% depending on the adequacy of mechanical removal, use of adjuvants, anatomical localization, and epigenetic signatures from H3.3 histone A (H3F3A)-mutated stromal cells [23,24,25]. Malignant transformation is rare [26,27], and histologically, transformed lesions show high-grade sarcomatous features, often losing the classic giant cell pattern [26]. Pulmonary metastases occur in 1 to 4% of cases with the metastatic mechanism remaining unclear, but it may involve stromal cell clusters entering venous circulation [28].

The identification of recurrent H3F3A mutations in neoplastic stromal cells has revolutionized the conceptualization of GCTB [29,30,31,32], providing a unifying pathogenic mechanism and establishing immunohistochemistry for mutation H3.3 G34W as a decisive diagnostic tool [29,30,31,32]. In fact, the identification of driver mutations in H3F3A conclusively positioned the mononuclear stromal cell as the true neoplastic component [33].

From a clinical standpoint, GCTB typically arises in the epiphysis of long bones, especially the distal femur, proximal tibia, and distal radius (these three locations contribute to approximately 60% of cases), though axial skeleton involvement presents unique management challenges [34,35,36]. Lesions near joints often present late because of slow cortical breakthrough and joint-sparing expansion into cancellous bone [7,13,37]. GCTB is uncommon in the craniofacial region, and its clinical presentation can complicate diagnosis. Because the jaw bones can harbor a wide range of cysts and tumors of different origins, lesions lacking odontogenic features require careful investigation. Tumor biomarkers may assist surgeons in developing an appropriate treatment plan and predicting disease prognosis [38,39,40].

A hallmark of the disease is aggressive osteolysis driven by the overexpression of receptor activator of nuclear factor kappa B ligand (RANKL) in stromal cells, resulting in significant bone destruction and functional impairment [41,42,43,44]. Interestingly, GCTB represents a paradigm of paracrine neoplasia wherein neoplastic stromal cells orchestrate the recruitment and fusion of osteoclast precursors via excessive RANKL expression, leading to profound osteolysis [41,44,45,46,47]. Accordingly, the tumor’s characteristic giant cells function as reactive effectors rather than neoplastic elements and are predominantly of macrophage origin (CD68-positive, CD163-negative), and their phenotype aligns more with osteoclast-like cells in bone lesions than with macrophage polykaryons in extraosseous lesions [48].

From a pathological perspective, GCTB represents a model of tumor–microenvironment interaction, where neoplastic cells utilize osteoclastogenic signaling to remodel bone for their own expansion [44,49,50,51]. Understanding this biology is essential to modern clinical management, particularly in the era of denosumab, which directly targets RANKL and reshapes tumor morphology [42,52,53].

Proposed risk factors for the development of this neoplasm include previous trauma, Paget’s disease of bone, genetic predisposition, and radiation exposure. Although these associations are not fully understood, they suggest that both environmental and genetic factors may contribute to tumor pathogenesis [26,54]. In this context, the aim of this narrative review is to synthesize current evidence on the biology, pathophysiology, and histopathology of GCTB.

## 2. Search of Existing Evidence

A structured narrative review methodology was adopted to ensure rigor and reproducibility. The literature was identified through a search in PubMed/MEDLINE, Scopus and Web of Science. We also used the WHO Classification of Soft Tissue and Bone Tumors (5th edition, 2020) [22]. Search terms included: “giant cell tumor of bone”, “H3F3A”, “osteoclastogenesis”, “RANKL”, “giant cell neoplasm”, “denosumab”, “histopathology”, “epigenetic regulation”, and “bone tumor”.

Inclusion criteria included: English-language, peer-reviewed studies; pertinence to biology, pathophysiology, histopathology, diagnosis, imaging, or treatment; and publication date of 1990–2025, with a preference for the post-2010 molecular literature. On the other hand, exclusion criteria included: case reports without mechanistic insights; non-skeletal giant cell lesions; pediatric epiphyseal cystic lesions; and articles focusing on giant cell-rich sarcomas (except for differential diagnosis).

The content was analyzed and involved the following thematic areas: molecular biology and genetics; tumor microenvironment; osteoclastogenesis; histopathology; radiology and clinical behavior; and therapeutic implications.

The key references are described in Appendix A (Table A1).

## 3. Bone Homeostasis in Normal Physiology

Bone is a rigid and dynamic tissue, composed of living bone tissue cells, that undergoes continuous remodeling throughout one’s life to maintain structural integrity, mineral homeostasis, and mechanical function [55,56,57]. This process is tightly regulated by osteoclasts, osteoblasts, and osteocytes. Osteoclasts, derived from monocyte–macrophage lineage precursors, mediate bone resorption, whereas osteoblasts, originating from mesenchymal cells, are responsible for bone matrix synthesis and mineralization [58]. Osteocytes, terminally differentiated from osteoblasts embedded within the mineralized matrix, function as mechanosensors and orchestrators of bone remodeling through paracrine signaling [55,56,57,58].

A central regulatory axis in bone homeostasis is the RANKL–osteoprotegerin (OPG) pathway. RANKL, expressed by osteoblasts, binds to its receptor RANK on osteoclast precursors, promoting their differentiation, activation, and survival. OPG, a receptor secreted by osteoblast lineage cells, antagonizes RANKL and limits osteoclastogenesis [57,59]. The balance between RANKL and OPG is influenced by systemic hormones (such as parathyroid hormone, estrogen and vitamin D) and local cytokines, ensuring coupling between bone resorption and formation [59].

Physiological bone remodeling is further modulated by signaling pathways such as Wnt/β-catenin, transforming growth factor-β (TGF-β), and bone morphogenetic proteins (BMPs), which regulate osteoblast differentiation and activity [60]. The disruption of these finely tuned mechanisms results in pathological bone loss or excessive resorption, as exemplified in GCTB [61].

## 4. Molecular Biology of Giant Cell Tumor of Bone and Tumorigenesis

GCTB is now recognized as a genetically defined neoplasm driven by recurrent mutations in H3F3A, the gene encoding the histone variant H3.3 [62,63,64]. This discovery reshaped the conceptualization of the tumor from a benign osteoclastic proliferation to a clonal neoplasm of mesenchymal stromal cells [14,15,65,66].

The H3F3A mutation present in most tumors (~90%) consists of p.G34W mutations [30,45,67,68]. Less common mutations are G34V, G34L, G34M, G34R and G34K [45,69,70]. These mutations affect glycine 34, a critical residue near lysine 36 (H3K36), a major regulatory site for transcription, splicing, and chromatin structure [71,72,73]. As a consequence, there is a disruption of the interaction with H3K36 methyltransferase hypomethylation (SETD2), perturbation of the H3K36 landscape, alterations in the transcription of osteoblastic lineage genes, upregulation of RANKL expression, altered nucleosome positioning and the induction of a phenotype resembling defective osteoblastic differentiation [29,73,74].

The aberrant epigenetic state defines the neoplastic stromal cell, the only proliferative cell population in GCTB [50,73,75]. Transcriptomic studies show resemblance to immature osteoblast precursors, limited osteoid production and the absence of terminal osteoblastic markers [44,76,77,78]. These cells orchestrate the recruitment of reactive osteoclast-type giant cells, which constitute the hallmark histological feature but are not neoplastic cells [15,41,44,79].

Neoplastic stromal cells are mononuclear and represent only a small proportion of the total tumor volume, yet they orchestrate the tumor phenotype [15,43,65,79]. These cells originate from mesenchymal stromal lineage; have H3F3A mutations; and secrete high levels of RANKL, colony-stimulating factor 1 (CSF-1) and matrix metalloproteinases (MMPs) that recruit monocytes and promote giant cell formation [42,50,65,66,80]. Their transcriptional profile resembles osteoblastic precursors but with impaired maturation [44,79,81].

Circulating monocytes and tissue-resident macrophages are recruited from the bloodstream and local tissues and differentiate into osteoclast precursors under the influence of CSF-1. These precursor cells are genetically normal and do not exhibit intrinsic molecular alterations [79,82,83,84,85]. Subsequent stimulation by RANKL induces the fusion of monocyte-derived precursors, leading to the formation of multinucleated giant cells that function as mature osteoclasts and are responsible for osteoclastic bone resorption [79,86,87].

As previously described, the H3F3A mutation plays a pivotal role in the pathogenesis of GCTB [5,67,68]. This mutation disrupts normal histone interactions with the epigenetic regulatory machinery, effectively “locking” stromal cells in an undifferentiated progenitor state. This arrested state fosters a permissive microenvironment for osteoclastogenesis, driving the recruitment and activation of osteoclast precursors and contributing to the locally aggressive behavior of the tumor [45,50,65,74,88].

Neoplastic stromal cells in GCTB overproduce RANKL, while osteoprotegerin (OPG), the physiological decoy receptor, is underexpressed. This imbalance drives osteoclast precursor cells, bearing the RANK receptor, to fuse and form multinucleated giant cells, resulting in extensive bone resorption and the locally aggressive nature of the tumor [51,89,90].

GCTB was traditionally treated with surgery, which is often associated with significant morbidity. The introduction of denosumab, a monoclonal antibody that inhibits RANKL, represents a therapeutic advance by allowing for, in many cases, tumor volume reduction and the preservation of anatomical function, with less functional impact [42,91,92]. Denosumab induces histological changes in GCTB. Pharmacological treatment can result in the near-complete elimination of osteoclast-like giant cells and a substantial reduction in the proliferative stromal cell population. These regions are subsequently replaced by dense fibro-osseous tissue and newly formed woven bone, characterized by increased mineral density and a disorganized collagen matrix. Collectively, these findings indicate that denosumab not only suppresses osteoclast-mediated bone resorption but also allows for ossification and structural remodeling within the tumor [13,52,53,93,94,95].

Beyond RANKL inhibition, other molecular factors contribute to tumor behavior: CSF-1 promotes the recruitment and survival of macrophages and osteoclast precursors [84,96,97], while matrix metalloproteinases facilitate local invasion and bone remodeling [98,99,100]. Vascular endothelial growth factor (VEGF) is frequently upregulated, correlating with increased vascular density [101,102,103], and programmed death ligand 1 (PD-L1) expression has been demonstrated, suggesting potential immune checkpoint interactions. Together, these mechanisms underscore the complex microenvironment driving GCTB progression and therapeutic response [104,105,106].

Collectively, these microenvironmental drivers have important therapeutic implications that extend beyond conventional RANKL inhibition. While agents such as denosumab can disrupt osteoclastogenesis, they do not directly target the H3F3A-mutant neoplastic stromal cells or other signaling networks embedded within this pro-tumorigenic niche [64,107]. The persistent activation of pathways involving CSF-1, VEGF, matrix metalloproteinases, and immune checkpoint molecules such as PD-L1 may contribute to residual tumor activity, adaptive resistance, and disease recurrence following treatment withdrawal [101,102,106,108]. These observations provide a biological rationale for emerging therapeutic strategies that integrate osteoclast inhibition with approaches aimed at the epigenetic reprogramming of stromal cells or modulation of tumor–immune interactions. Accordingly, a deeper understanding of how sustained osteoclast suppression reshapes the local epigenetic, vascular, and immune landscape is essential for optimizing treatment sequencing, minimizing long-term risks, and advancing combination therapies in H3F3A-driven giant cell tumor of bone.

## 5. Pathophysiology: The Osteoclastogenic Microenvironment

GCTB is a paradigmatic example of a neoplasm that manipulates normal bone homeostasis to promote its own local expansion. The tumoral stromal cell drives this process through the pronounced dysregulation of the RANK–RANKL–OPG signaling axis. Neoplastic stromal cells express RANKL at markedly higher levels than normal osteoblasts, producing a local microenvironment that favors osteoclastogenesis and bone destruction [13,77,109,110,111]. Concomitantly, the expression of the decoy receptor OPG is relatively reduced in these lesions, which removes a physiologic brake on RANKL activity and further shifts the balance toward osteoclast differentiation and activation [51,89,90].

Circulating RANK-positive mononuclear precursors are chemoattracted to the tumor microenvironment, where exposure to stromal-derived RANKL (and other supportive factors) drives their differentiation and fusion into large, multinucleated osteoclast-like giant cells that are the histologic hallmark of GCTB [51,89,90]. These reactive giant cells, while not neoplastic themselves, are the principal effectors of the aggressive osteolysis seen in GCTB. Their resorptive activity is mediated by multiple complementary mechanisms: the secretion of the cysteine protease cathepsin K, acidification of resorption lacunae via proton pumps to dissolve mineral, and localized release of MMPs and other proteases that degrade the organic bone matrix. Together with the physical disruption of bone architecture, these processes produce the characteristic expansile lytic lesions and frequently result in cortical thinning, pathologic fracture, and rapid local progression [80,112,113].

The net result is a self-sustaining cycle in which neoplastic stromal cells recruit and activate osteoclast lineage cells that, in turn, create space for continued stromal proliferation and tumor expansion. This pathogenic model has important translational implications: interrupting RANKL-mediated signaling can suppress osteoclast formation and activity and thereby reduce tumoral bone resorption and mass effect. Clinically, this mechanistic insight underpins targeted therapeutic strategies aimed at neutralizing RANKL and modulating the stromal–osteoclast axis.

In addition to neoplastic stromal cells and multinucleated giant cells, intermediary histiocytic mononuclear cells play a key modulatory role within the GCTB microenvironment. These cells actively contribute to tumor-associated osteolysis through the secretion of a broad array of soluble mediators, including pro-osteoclastogenic cytokines such as interleukin-6 (IL-6) and tumor necrosis factor-α (TNF-α); chemotactic and differentiation factors such as macrophage colony-stimulating factor (M-CSF) and chemokine C-C motif ligand 20 (CCL20); and fusion-related molecules, including dendritic cell-specific transmembrane protein (DC-STAMP) [14,15,44,49,114,115]. Collectively, these factors enhance the recruitment, survival, and fusion of osteoclast precursors, reinforcing a self-perpetuating positive feedback loop that sustains excessive osteoclastogenesis and bone resorption.

**Figure 1:**
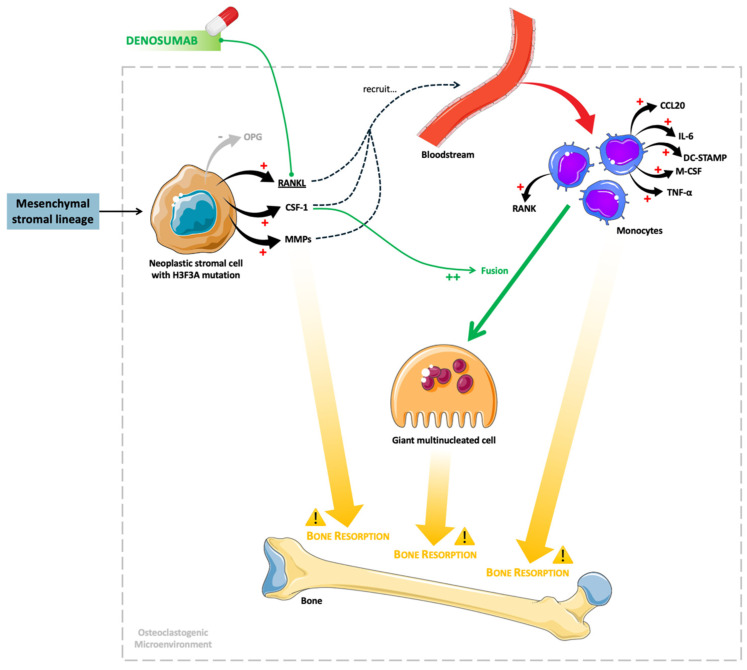
The mechanisms underlying giant cell tumor of bone, as well as the site of action of denosumab. Legend: CCL20, chemokine C-C motif ligand 20; CSF-1, colony-stimulating factor 1; DC-STAMP, dendritic cell-specific transmembrane protein; H3F3A, H3.3 histone A; IL-6, interleukin-6; M-CSF, macrophage colony-stimulating factor; MMPs, matrix metalloproteinases; OPG, osteoprotegerin; RANK, receptor activator of nuclear factor kappa B; RANKL, receptor activator of nuclear factor kappa B ligand; TNF-α, tumor necrosis factor alpha. Parts of this figure were drawn using pictures from Servier Medical Art by Servier (https://smart.servier.com), which is licensed under Attribution 4.0 International (Creative Commons CC BY 4.0).

In this context, the destructive capacity of GCTB is multifactorial and reflects the convergence of several pathogenic mechanisms: (i) direct osteoclastic bone resorption mediated by multinucleated giant cells; (ii) stromal cell-driven osteolysis through sustained RANKL-rich signaling; (iii) the degradation of osteoid and the extracellular matrix via the overexpression of matrix metalloproteinases; and (iv) progressive expansion within cancellous bone leading to cortical thinning, breach, and eventual extension into adjacent soft tissues. These biological processes account for the characteristic radiological appearance of GCTB, which typically manifests as an expansile, lytic, eccentric lesion lacking matrix mineralization [34,116]. Figure 1 shows the mechanisms underlying GCTB, as well as the site of action of denosumab.

## 6. Gross Pathology and Histopathology

Histopathological evaluation is fundamental to the diagnosis of GCTB, as the lesion exhibits a characteristic microscopic appearance but must be carefully distinguished from several giant cell-rich mimics. The pathology reflects the tumor’s biological organization: neoplastic stromal cells drive the recruitment and differentiation of osteoclast-like giant cells, leading to the archetypal morphological pattern.

The histopathological features of GCTB closely mirror its underlying biological organization. Neoplastic stromal cells orchestrate the recruitment, differentiation, and activation of osteoclast lineage cells, resulting in the characteristic giant cell-rich morphology that defines the lesion [15,80]. This intimate interaction between neoplastic and reactive cellular components underpins both the histologic appearance and aggressive osteolytic behavior of GCTB.

Macroscopically, GCTB typically presents as a soft, friable, tan-brown mass, frequently containing areas of hemorrhage and cystic degeneration. The tumor most often arises in the metaphyseal–epiphyseal region of long bones and demonstrates an expansile growth pattern, with the progressive thinning or breach of the cortical bone and extension into adjacent soft tissues in advanced cases [8,16,37]. Tumor margins commonly show irregular penetration of the cortex, producing a characteristic “worm-hole” pattern of cortical erosion that reflects infiltrative local growth [34,117,118].

Histopathological examination remains the diagnostic cornerstone of GCTB [45,119]. At low magnification, the tumor is characterized by sheets of mononuclear stromal cells interspersed with numerous, evenly distributed osteoclast-type multinucleated giant cells, along with prominent hemorrhage and hemosiderin deposition. Notably, there is an absence of osteoid or chondroid matrix production, except in cases previously treated with denosumab, in which reactive bone formation may be observed [49,114]. At higher magnification, the mononuclear stromal cells display oval- to spindle-shaped nuclei, fine chromatin, inconspicuous nucleoli, and minimal cytologic atypia. Mitotic figures are frequently present but typically normal in morphology, consistent with the proliferative yet low-grade nature of the neoplastic stromal population. In contrast, the multinucleated giant cells are non-neoplastic, closely resemble physiologic osteoclasts, and usually contain 20 to 50 uniform nuclei that are morphologically similar to those of the surrounding stromal cells [14,42,49,65,120].

Immunohistochemistry provides valuable support for the diagnosis and differential diagnosis of GCTB. A hallmark feature is strong nuclear positivity for the H3.3 G34W mutant protein, which is restricted to neoplastic stromal cells and serves as a highly specific diagnostic marker [30,62,121]. The expression of special AT-rich sequence-binding protein 2 (SATB2) further supports the osteoblastic lineage of the stromal cells [122,123,124]. Markers such as CD68 and CD163 highlight the histiocytic and osteoclast lineage populations, particularly within the multinucleated giant cells and mononuclear precursors [48,81,125]. The proliferative index, as assessed by Ki-67, is variable but generally moderate, typically ranging from 5% to 20% [120,126,127].

Equally important is the absence of immunoreactivity for markers associated with histologic mimics. Negativity for H3F3B, mouse double minute 2 homolog (MDM2), cytokeratin, and cyclin-dependent kinase 4 (CDK4) assists in excluding chondroblastoma, osteosarcoma, metastatic carcinoma, and low-grade osteosarcoma, respectively [33,128,129,130,131,132]. Given the broad differential diagnosis of giant cell-rich bone lesions, the careful integration of histomorphology, immunohistochemistry, and clinical–radiological correlation is essential for accurate diagnosis.

## 7. The Management of Giant Cell Tumor of Bone and the Role of Denosumab

The recommended primary treatment for GCTB is surgical resection, whenever feasible, with the aim of achieving durable local control while preserving anatomical integrity and function. Intralesional curettage with or without local adjuvants (high-speed burring, phenol, argon beam coagulation, or polymethylmethacrylate cementation) is preferred for most appendicular lesions [133]. En bloc resection is reserved for select cases with extensive cortical destruction, soft tissue extension, or recurrent disease where intralesional surgery would result in unacceptable recurrence risk [133].

As previously described, denosumab has emerged as an important systemic therapy for GCTB based on its ability to inhibit osteoclast-like giant cells and suppress osteolysis [134]. It is primarily indicated for patients with unresectable tumors, relapsed or recurrent disease after surgery, or situations in which surgical resection would result in severe morbidity [134]. Denosumab leads to a rapid reduction in tumor-associated giant cells and decreased bone resorption and allows for progressive new bone formation [134]. The approved dosing regimen for denosumab in GCTB consists of 120mg administered subcutaneously every 4 weeks, with additional loading doses on days 8 and 15 of the first month [135]. Calcium and vitamin D supplementation is mandatory to reduce the risk of hypocalcemia [135]. While denosumab is effective in disease control, optimal treatment duration remains uncertain. Prolonged therapy has been associated with concerns including rebound hypercalcemia after discontinuation, atypical femoral fractures, osteonecrosis of the jaw, and potential effects on tumor biology [92]. Consequently, treatment with denosumab and associated therapies should be individualized to achieve the maximum tumor control while minimizing treatment-related morbidity and preserving function.

## 8. Post-Denosumab Histology

Denosumab therapy induces profound and characteristic histopathological alterations in GCTB, substantially modifying its conventional morphology [92,136,137]. By inhibiting RANKL-mediated osteoclastogenesis, denosumab results in a marked depletion or complete absence of multinucleated osteoclast-type giant cells. Concomitantly, there is prominent deposition of newly formed woven bone and osteoid, often arranged in irregular trabeculae, along with the development of sclerotic, matrix-rich areas that may simulate malignant bone-forming tumors, particularly osteosarcoma [94,120,136].

In parallel, the neoplastic stromal cell population undergoes notable phenotypic changes, becoming smaller, more uniform, and cytologically bland, with a reduction in cellularity and proliferative activity. These therapy-induced modifications can obscure the classic histologic features of GCTB and give rise to diagnostic challenges, as the abundant reactive bone formation may closely resemble osteoblastoma or fibro-osseous lesions. Consequently, accurate diagnosis in denosumab-treated cases requires careful correlation with clinical history, radiologic findings, and immunohistochemical confirmation of the underlying GCTB genotype, particularly through the demonstration of H3F3A (H3.3 G34W) mutant protein expression in residual stromal cells [62,67,138].

## 9. Treatment Algorithm Considerations and Special Scenarios

In most cases, denosumab is utilized as a secondary or adjunctive therapy following local recurrence after initial surgical management [139]. Its use in the neoadjuvant setting remains controversial, being restricted to uncommon scenarios in which tumor downsizing may facilitate the surgery [140].

For giant cell tumors of the spine, particularly vertebral body lesions where surgical resection could be associated with substantial neurological or mechanical morbidity, alternative local control strategies may be considered. Stereotactic body radiotherapy has emerged as a potential option in select cases, offering high-dose conformal radiation with the relative sparing of surrounding critical structures [141], being considered for unresectable vertebral GCTB, residual disease after incomplete surgery, or patients who are not surgical candidates [141]. Although conventional radiotherapy has historically been associated with concerns regarding malignant transformation, these modern techniques appear to offer improved local control with a more favorable safety profile, though long-term data remain limited [142].

A multidisciplinary approach integrating different medical specialties is essential to tailor treatment strategies, particularly in anatomically challenging or recurrent disease, promoting individualized care pathways in GCTB.

## 10. Conclusions and Perspectives

GCTB is recognized as a molecularly defined neoplasm driven by H3F3A-mutated stromal cells that orchestrate a highly specialized and profoundly osteoclastogenic tumor microenvironment. The identification of the H3.3 G34W mutation has transformed diagnostic practice, enabling discrimination from histologic mimics and establishing GCTB as a mesenchymal neoplasm with osteoblastic differentiation potential.

Neoplastic stromal cells exhibit marked epigenetic dysregulation, aberrant chromatin architecture, and altered transcriptional programs, which collectively result in the sustained overexpression of RANKL and the recruitment, differentiation, and activation of osteoclast lineage cells. The resulting multinucleated giant cells are reactive rather than neoplastic, yet they account for the tumor’s characteristic morphology and its locally aggressive osteolytic behavior.

Histopathology remains the gold standard to confirm the diagnosis, and its interpretation has evolved in the era of targeted therapy. Denosumab remodels the tumor microenvironment by affecting giant cells and inducing reactive bone formation, changes that may mimic osteoid-producing neoplasms and obscure classic histologic features. In this context, accurate diagnosis requires the careful integration of clinical history, radiologic findings, and immunohistochemical confirmation of the underlying H3F3A mutation, particularly in treated or recurrent lesions.

**Figure 2:**
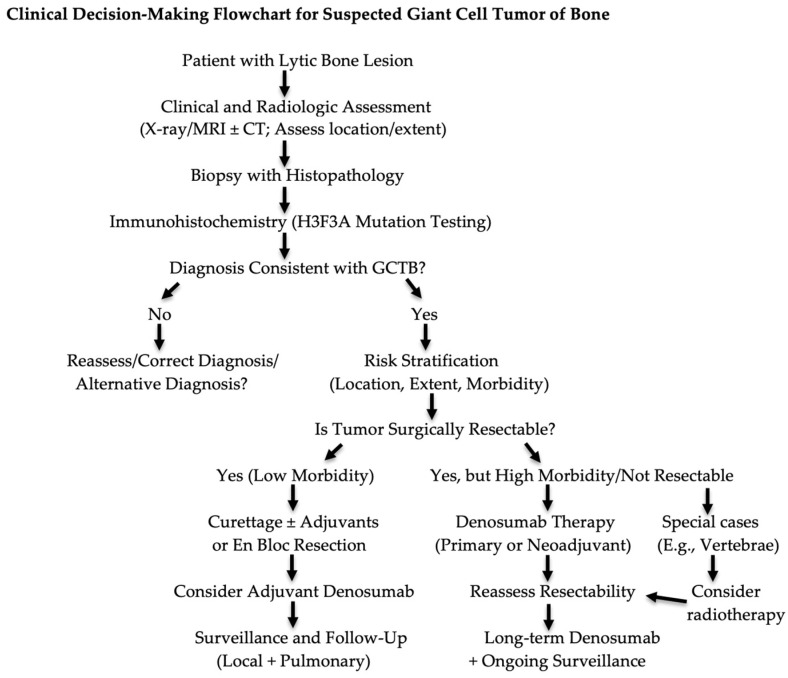
Clinical decision-making flowchart for suspected giant cell tumor of bone. Legend: CT, computed tomography; H3F3A, H3.3 histone A; MRI, magnetic resonance imaging.

Clinically, GCTB is characterized by locally aggressive bone destruction and a substantial risk of local recurrence while generally maintaining a favorable overall prognosis. Current management includes pharmacological treatment with denosumab, surgical approaches ranging from intralesional curettage to more extensive resection, radiotherapy for specific cases or combined strategies (Figure 2). Denosumab plays an important adjunctive role in tumor downstaging, neoadjuvant therapy, and the management of unresectable disease. Nevertheless, important unanswered questions remain regarding the long-term consequences of RANKL inhibition, including recurrence patterns following treatment cessation, the risk of malignant transformation, and the optimal duration and timing of therapy.

From a decision-making perspective, the use of denosumab requires careful patient selection and risk stratification. Although effective in suppressing osteoclast-mediated bone destruction, denosumab does not eradicate the H3F3A-mutant neoplastic stromal cells that drive tumor biology, which may contribute to disease persistence and recurrence after treatment discontinuation [21,64]. Prolonged exposure has been associated with increased rates of local recurrence following intralesional surgery, while abrupt cessation, particularly in younger patients, may lead to rebound osteoclast activation and hypercalcemia [143]. In addition, denosumab-induced histological changes, such as the depletion of giant cells and formation of woven bone, can obscure residual tumor and complicate both surgical margins and pathological interpretation. Rare but clinically relevant reports of sarcomatous transformation further underscore the importance of close radiological, biochemical, and histopathological monitoring [53,144]. Collectively, these considerations support a multidisciplinary, individualized approach in which treatment duration, sequencing with surgery, and discontinuation strategies are predefined to balance tumor control with long-term safety.

In parallel, accurate diagnosis remains critical given the wide differential spectrum of giant cell-rich bone lesions. Several benign and malignant entities, including aneurysmal bone cyst, chondroblastoma, giant cell-rich osteosarcoma, brown tumor of hyperparathyroidism, and tenosynovial giant cell tumor, may closely mimic GCTB in terms of morphology alone [145]. In this context, the integration of mutation-specific immunohistochemistry, particularly H3F3A G34W, has become an essential diagnostic tool, allowing for the reliable discrimination of GCTB from its mimics. Additional markers such as H3F3B K36M, SATB2, MDM2/CDK4, and cytokeratin provide further practical utility in resolving challenging cases, especially in post-denosumab specimens where treatment-induced histological alterations may obscure classic features [30,45,64,67,68,120]. Incorporating immunohistochemical and molecular markers into routine diagnostic workflows enhances diagnostic precision and supports appropriate therapeutic decision-making.

Future research priorities include elucidating the clonal evolution and intratumoral heterogeneity of neoplastic stromal cells; identifying molecular or histologic biomarkers predictive of recurrence, metastasis, and malignant transformation; defining the long-term safety and biological impact of denosumab; and developing stromal cell-specific targeted therapies beyond RANKL blockade. Particular attention should be directed toward exploiting the epigenetic vulnerabilities created by the H3F3A mutation as potential therapeutic targets. Also, future research should move beyond descriptive molecular profiling toward integrative, data-driven frameworks capable of informing clinical decision-making. In this context, artificial intelligence and machine learning approaches offer particular promise for risk stratification by combining histopathological features, radiologic patterns, and genomic alterations such as H3F3A mutation status [146,147]. Such models could improve the prediction of local recurrence, malignant transformation, and therapeutic response, enabling personalized surveillance and treatment strategies [146,147]. Prospective, multicenter datasets will be essential to validate these approaches and ensure their applicability across diverse populations [146,147].

Based on current molecular and biological evidence, a hypothetical “H3F3A-driven epigenetic therapy model” can be outlined, suggesting that durable disease control in giant cell tumor of bone may require therapeutic strategies that extend beyond osteoclast suppression and directly address the mutant neoplastic stromal compartment. Within this conceptual framework, RANKL inhibition primarily mitigates the downstream mechanisms of bone destruction, whereas future approaches may focus on reversing aberrant chromatin states induced by H3F3A mutations, restoring osteoblastic differentiation programs, or modulating the immune and vascular components of the tumor microenvironment. Epigenetic therapies, stromal-targeted agents, and immune-based interventions could ultimately be integrated with osteoclast inhibition to achieve more sustained biological control. This framework positions giant cell tumor of bone as a prototype of mutation-driven, microenvironment-dependent bone neoplasia and offers a translational rationale for next-generation combination therapeutic strategies [73,148,149].

This narrative review has inherent limitations due to its non-systematic design, which may introduce selection and publication bias and precludes quantitative synthesis; despite the use of a structured search strategy, the predominance of retrospective clinical series and small observational cohorts in the GCTB literature makes the selection and interpretation of studies particularly susceptible to publication and reporting biases. In fact, the available evidence on giant cell tumor of bone is heterogeneous and largely derived from retrospective studies, small clinical series, and experimental models, limiting comparability and generalizability. In addition, the interpretation of histopathologic features, particularly in denosumab-treated lesions, remains partly subjective, and long-term data on recurrence, malignant transformation, and optimal therapeutic strategies are still limited. Future systematic reviews, ideally supported by a more standardized reporting of outcomes, harmonized diagnostic criteria, and prospective multicenter datasets, may be better positioned to reduce these sources of bias, enable more robust comparative analyses, and potentially allow for meaningful quantitative synthesis or meta-analytic approaches. Moreover, future studies should be designed with greater methodological rigor, including well-structured prospective cohorts and, when feasible, randomized controlled trials. Such improvements in study quality would generate more robust and comparable data, ultimately enabling high-quality systematic reviews and meaningful meta-analyses that can better inform clinical practice.

In summary, GCTB lies at the intersection of bone biology, epigenetics, and tumor–microenvironment interactions. Advances in molecular pathology and targeted therapy have markedly improved diagnostic precision and clinical management; however, fully translating biological insights into durable, mechanism-based therapeutic strategies remains an ongoing challenge.

## Data Availability

All data generated or analyzed during this study are included in the manuscript.

## Abbreviations

The following abbreviations are used in this manuscript:

BMPs	Bone morphogenetic proteins
CCL20	Chemokine C-C motif ligand 20
CD	Cluster of differentiation
CD4	Cyclin-dependent kinase 4
CSF-1	Colony-stimulating factor 1
CT	Computed tomography
DC-STAMP	Dendritic cell-specific transmembrane protein
GCTB	Giant cell tumor of bone
H3F3A	H3.3 histone A
H3F3B	H3.3 histone B
IL	Interleukin
M-CSF	Macrophage colony-stimulating factor
MDM2	Mouse double minute 2 homolog
MMPs	Matrix metalloproteinases
MRI	Magnetic resonance imaging
OPG	Osteoprotegerin
PD-L1	Programmed death ligand 1
RANK	Receptor activator of nuclear factor kappa B
RANKL	Receptor activator of nuclear factor kappa B ligand
SATB2	AT-rich sequence-binding protein 2
SETD2	H3K36 methyltransferase hypomethylation
TNF-α	Tumor necrosis factor alpha
TGF-β	Transforming growth factor-β
VEGF	Vascular endothelial growth factor
WHO	World Health Organization

## Appendix A

**Table A1:** Key references of giant cell tumor of bone (GCTB).

Theme	Ref.	Title	PMID	Type
Epidemiology	[1]	Incidence and demographics of giant cell tumor of bone in The Netherlands: First nationwide Pathology Registry Study	29987945	Population-based registry (NL)
Epidemiology	[2]	Epidemiology of benign giant cell tumor of bone in the Chinese population	30148063	Population-based (China)
Epidemiology	[4]	Population-based study of giant cell tumor of bone in Sweden (1983–2011)	27060625	Longitudinal cohort (SE)
Clinical Cohorts	[9]	Giant cell tumor of bone: A single center study of 115 cases	35242511	Single-center outcomes
Molecular Pathogenesis	[33]	Distinct H3F3A and H3F3B driver mutations define chondroblastoma and giant cell tumor of bone	24162739	Foundational genetics
Molecular Pathogenesis	[29]	Histone H3.3 mutation in giant cell tumor of bone: an update in pathology	31748824	Modern pathology update
Molecular Pathogenesis	[71]	H3.3 G34W promotes growth and impedes differentiation of osteoblast-like mesenchymal progenitors in GCTB	32967858	Functional mechanism
Epigenetics	[74]	Globally altered epigenetic landscape and delayed osteogenic differentiation in H3.3-G34W-mutant GCTB	33110075	Epigenome profiling
Epigenetics	[73]	Mutation-driven epigenetic alterations as a defining hallmark of cartilaginous tumours, GCTB and chondroblastoma	31728625	Review of epigenetics
Diagnostics	[30]	H3F3A (Histone 3.3) G34W immunohistochemistry: a reliable marker defining benign and malignant in GCTB	28505000	Diagnostic IHC
Diagnostics	[31]	Reliability and role of mutation-specific H3F3A (Histone 3-3) G34W immunohistochemistry to differentiate GCTB from its clinicoradiologic and histologic mimics: an institutional study	34347625	Validation study
Diagnostics	[69]	H3F3A G34 mutation DNA sequencing and G34W immunohistochemistry analysis in 366 cases of GCTB and other bone tumors	33021329	Large series
Biomarkers/Therapeutic Targets	[77]	Biomarkers and therapeutic targets in GCTB: a comprehensive review	40532838	State-of-the-art review
Biomarkers	[32]	Significance of biogenetic markers in GCTB differentiation and prognosis: A narrative review	39795567	Narrative review
Biomarkers	[64]	Histological, immunohistochemical and H3F3A mutation analyses	29206281	Clinicopathological study
Malignancy/Transformation	[26]	Malignancy in GCTB: a review of the literature	30935298	Systematic review
Malignancy/Transformation	[27]	Malignancy in GCTB: analysis of an open-label phase 2 study of denosumab	33482769	Clinical analysis
Malignancy/Genetics	[138]	Profiling of three H3F3A-mutated and denosumab-treated GCTB points to diverging pathways during progression and malignant transformation	33707617	NGS profiling
Malignancy/Genetics	[6]	Malignant GCTB: clinicopathologic series highlighting genetic differences compared with conventional, atypical, and metastasizing conventional tumors	40077813	Comparative genetics
Treatment/Concepts	[21]	Current concepts in the treatment of GCTB: an update	38668060	Therapeutic overview
Denosumab/Trials	[135]	Denosumab in patients with GCTB: multicentre, open-label, phase 2 study	31704134	Pivotal phase II
Denosumab/Real-World	[42]	Denosumab in GCTB: multidisciplinary medical management based on pathophysiological mechanisms and real-world evidence	35565419	Clinical practice review
Denosumab/Surgery	[91]	The role of denosumab for surgical outcomes in GCTB: a systematic review	33809979	Surgery interface
Denosumab/Spine	[94]	Denosumab treatment of GCTB in the spine induces woven bone formation	40390810	Spinal cases
Microenvironment/Angiogenesis	[47]	Stromal cell–derived CCL20 promotes tumor progression and osteolysis in GCTB	30537747	Chemokine axis
Microenvironment/VEGF	[103]	Single-cell RNA sequencing reveals differential expression of EGFL7 and VEGF in GCTB and osteosarcoma	35695550	scRNA-seq
Immune/PD-L1	[104]	Prognostic role of PD-L1 and immune-related gene expression profiles in GCTB	32377818	Prognostic immunology
Immune/PD-L1 and Denosumab	[105]	Tumor microenvironment in GCTB: evaluation of PD-L1 expression and SIRPα infiltration after denosumab treatment	34285260	Immune changes post-therapy
Immune/Checkpoint Review	[106]	PD-1/PD-L1 immune checkpoint in bone and soft tissue tumors (Review)	39989606	Review—ICI context
Emerging Therapies	[75]	Histone deacetylase inhibitors as a therapeutic strategy to eliminate neoplastic stromal cells from GCTB	36230631	Preclinical HDACi
Paracrine Signaling	[88]	Aberrant paracrine signalling for bone remodelling underlies the mutant histone-driven GCTB	361382226	Mechanistic study
Translational Overview	[45]	Molecular pathological insights into tumorigenesis and progression of GCTB	40092569	Up-to-date synthesis
